# Supplementary material for: Photostimulation of brain lymphatics in male newborn and adult rodents for therapy of intraventricular hemorrhage
Source: Nat Commun. 2023 Sep 29;14:6104. doi: 10.1038/s41467-023-41710-y (PMC10541888; doi:10.1038/s41467-023-41710-y)
Supplement: Supplementary file 1 — Supplementary Information [file 41467_2023_41710_MOESM1_ESM.pdf]

Photostimulation of brain lymphatics in male newborn and adult rodents for therapy of intraventricular hemorrhage

Dongyu Li[1,2#], Shaojun Liu[1#], Tingting Yu[1*], Zhang Liu[1], Silin Sun[1], Denis Bragin[3,4], Alexander Shirokov[5,6], Nikita Navolokin[6,7], Olga Bragina[3], Zhengwu Hu[1,2], Jürgen Kurths[6,8,9,10], Ivan Fedosov[6], Inna Blokhina[6], Alexander Dubrovski[6], Alexander Khorovodov[6], Andrey Terskov[6], Maria Tzoy[6], Oxana Semyachkina-Glushkovskaya [6,8*], Dan Zhu[1*]

[1] Britton Chance Center for Biomedical Photonics - MoE Key Laboratory for Biomedical Photonics, Wuhan National Laboratory for Optoelectronics - Advanced Biomedical Imaging Facility, Huazhong University of Science and Technology, Wuhan, Hubei 430074, China.

[2] School of Optical Electronic Information, Huazhong University of Science and Technology, Wuhan, Hubei 430074, China.

[3] Lovelace Biomedical Research Institute, Albuquerque, NM 87108, USA.

[4] Department of Neurology University of New Mexico School of Medicine, Albuquerque, NM 87131, USA.

[5] Institute of Biochemistry and Physiology of Plants and Microorganisms, Russian Academy of Sciences, Prospekt Entuziastov 13, Saratov 410049, Russia.

[6] Saratov State University, Astrakhanskaya str., 83, Saratov, 410012, Russia.

[7] Saratov State Medical University, B. Kazachya str., 112, Saratov, 410012, Russia

[8] Physics Department, Humboldt University, Newtonstrasse 15, 12489 Berlin, Germany.

[9] Potsdam Institute for Climate Impact Research, Telegrafenberg A31, 14473 Potsdam, Germany.

[10] Sechenov First Moscow State Medical University, Bolshaya Pirogovskaya 2, building 4, 119435 Moscow, Russia

[#] These authors contributed equally to this work.

Corresponding authors: Dan Zhu, E-mail: dawnzh@mail.hust.edu.cn

Semyachkina-Glushkovskaya Oxana, E-mail: glushkovskaya@mail.ru

Ting-Ting Yu, E-mail: yutingting@hust.edu.cn

This file contains the following Supplementary figures and tables:

Supplementary Figure 1 - Schematic illustration of PS stimulation of RBCs clearance from mouse brain.

Supplementary Table 1 - Temperature (°C) at the external surface of skull and the brain cortex before and after PS.

Supplementary Figure 2 – The histological analysis (H&E) of the cortex before and after PS course 3 J/cm^2; 6 J/cm^2; 9 J/cm^2; 18 J/cm^2 and 27 J/cm^2 (on the skull surface) in adult mice.

Supplementary Table 2 – GNRs accumulation rate (arb. units) in dcLN in healthy adult mice before and after PS.

Supplementary Table 3 –GNRs level (μg/g tissue) in dcLN in healthy adult mice before and after PS.

Supplementary Figure 3 – Detection of effective time for fluorescent imaging of EBD (5%, 5 μL) accumulation in dcLNs after dye injection into the right lateral ventricle.

Supplementary Table 4 –EBD fluorescent intensity (arb. units) in dcLN in healthy adult mice before and after PS.

Supplementary Figure 4 - Scheme of the optical setup for measuring the mouse scalp and skull transmittance level for 1267 nm QD semiconductor laser.

Supplementary Figure 5 - The changes in the cerebral blood flow (CBF) detected by laser speckle contrast imaging before and after PS in single dose 9 J/cm^2 in adult healthy mice.

Supplementary Figure 6 – The changes in the CBF detected by laser speckle imaging in the sham group and after photodynamic ablation of MLVs in the Visudyne+Laser group.

Supplementary Figure 7 – The histological analysis (H&E) of the cortex before and after the PS course 4 J/cm^2 in newborn rats.

Supplementary Table 5 - Temperature (°C) at the external surface of skull and the brain cortex before and after PS in newborn rats.

Supplementary Figure 8 – 2D confocal images of fresh brains illustrating size of the right lateral ventricle (three projections) in 4-day old newborn rats in the control (sham) group, 3 days and 11 days after IVH without and after PS-course 4 J/cm^2.

Supplementary Figure 9 – Schematic illustration of time of performance of behavior tests in pups.

Supplementary Figure 10 – Therapeutic effects of the PS-9 J/cm^2 course in adult mice with IVH.

Supplementary Figure 11 – The PS effects on the locomotor and memory functions.

Supplementary Figure 12 – Schematic illustration of MLV diameter distribution calculation.

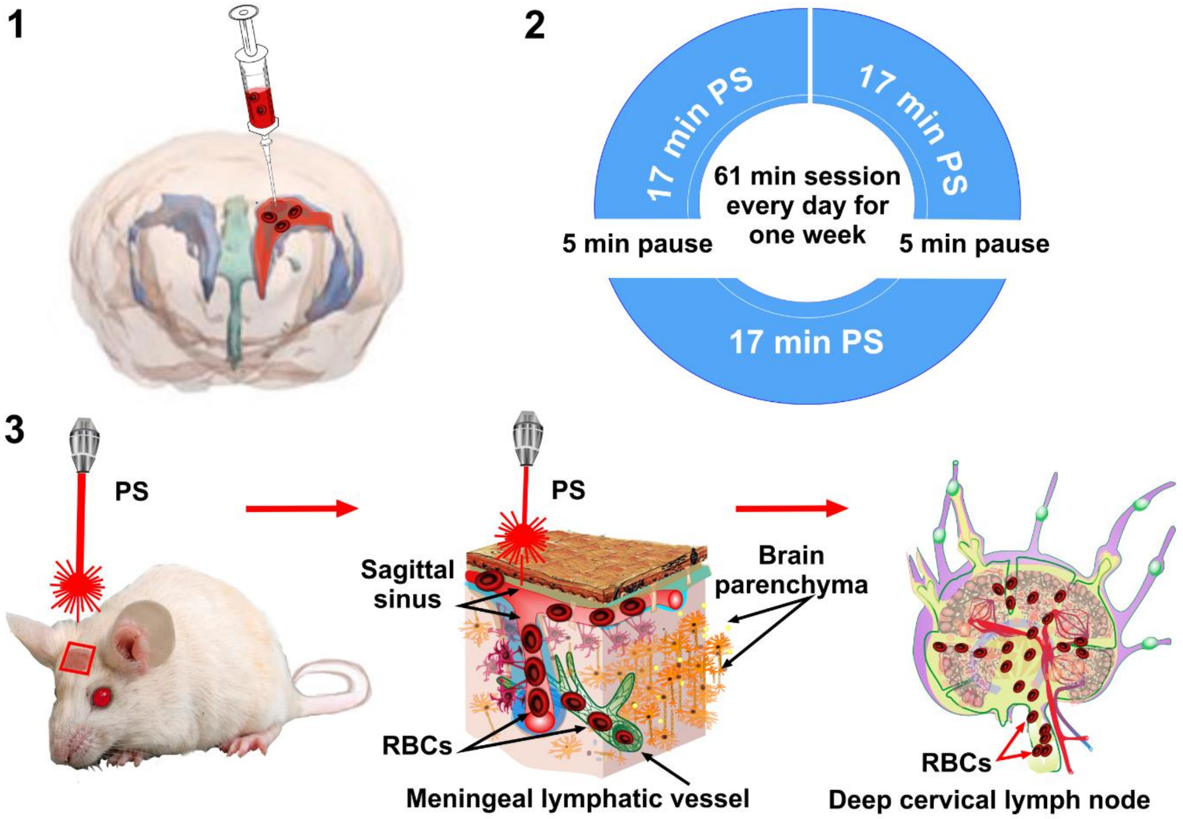

Supplementary Figure 1 - Schematic illustration of PS stimulation of RBCs clearance from mouse brain. (1) The autologous arterial blood was injected into the right lateral ventricle to induce IVH. (2) 3 days after a surgical procedure, transcranial PS (1267 nm, 63 J/cm^2) of the parietal cortex was performed every day for one week under inhalation anesthesia (1% isoflurane at 1 L/min N_2O/O_2 − 70:30) using the sequence of 17 min – irradiation, 5 min – pause, 61 min in total. (3) PS stimulates the RBCs clearance from the brain via MLVs into dcLNs.

Supplementary Table 1 - Temperature (°C) at the external surface of skull and the brain cortex before and after PS

Number of mouse/ Thermocouple positioning	1	2	3	4	5	6	7	8	9	10	Mean	SEM
no PS												
The skull external surface	35.49	35.57	35.68	35.74	36.46	35.31	36.26	36.15	35.84	35.71	**35.82**	**0.11**
Under the skull on the cortex surface	37.25	37.31	37.18	37.28	37.29	37.11	36.27	37.23	37.32	37.26	**37.15**	**0.10**
3 J/cm^2 PS												
The skull external surface	35.93	36.06	35.97	36.02	36.04	35.89	36.09	36.05	36.13	35.15	**35.93**	**0.09**
Under the skull on the cortex surface	36.98	37.13	37.51	36.91	37.09	37.08	37.02	37.12	37.11	36.95	**37.09**	**0.05**
6 J/cm^2 PS												
The skull external surface	35.89	36.39	36.29	35.57	35.52	35.88	36.11	35.98	35.92	36.08	**35.96**	**0.09**
Under the skull on the cortex surface	37.51	36.42	37.21	36.53	37.41	36.25	37.94	37.37	37.55	37.91	**37.21**	**0.19**
9 J/cm^2 PS												
The skull external surface	36.28	36.15	36.21	36.21	36.05	35.63	36.07	36.52	35.71	35.33	**36.02**	**0.11**
Under the skull on the cortex surface	37.00	37.71	37.12	37.49	36.71	36.55	36.81	37.22	37.58	37.37	**37.16**	**0.12**
18 J/cm^2 PS												
The skull external surface	36.48	36.44	36.38	36.52	36.49	36.55	36.62	36.67	36.71	36.38	**36.52**	**0.04**
Under the skull on the cortex surface	37.34	37.01	37.24	37.11	37.03	36.98	36.84	36.92	37.19	37.85	**37.15**	**0.09**
27 J/cm^2 PS												
The skull external surface	37.69	37.84	37.19	37.68	37.48	38.17	37.88	38.10	37.15	37.33	**37.65**	**0.11**
Under the skull on the cortex surface	37.43	37.39	36.55	37.17	37.02	37.08	37.55	37.72	37.19	36.64	**37.17**	**0.12**

	Wilcoxon signed rank test									
Thermocouple positioning	no PS & 3 PS		no PS & 6 PS		no PS & 9 PS		no PS & 18 PS		no PS & 27 PS	
	p-value	V	p-value	V	p-value	V	p-value	V	p-value	V
The skull external surface	**0.3076**	26	**0.388**	28	**0.1307**	16	**0.003675**	2	**0.003346**	0

The statistical significance in Supplementary Table 1 was assessed by Wilcoxon signed rank tests (n=10 mice in each group). The statistical tests involved two-sided analyses. Source data are provided as a Source Data file.

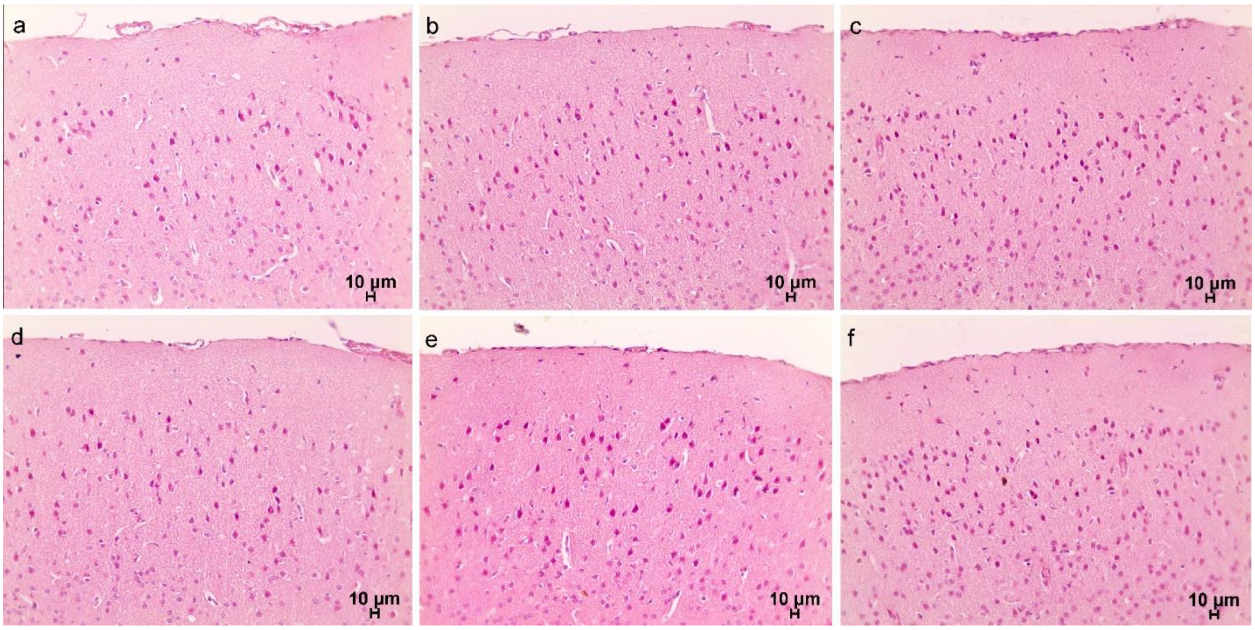

Supplementary Figure 2 – The histological analysis (H&E) of the cortex (**a**) before and after PS course (**b**) 3 J/cm^2; (**c**) 6 J/cm^2; (**d**) 9 J/cm^2; (**e**) 18 J/cm^2 and (**f**) 27 J/cm^2 (on the skull surface) in adult mice. Scale bar: 10 µm. 3 replicates.

Supplementary Table 2 – GNRs accumulation rate (arb. units) in dcLN in healthy adult mice before and after PS

Number of mouse/Time of observation of PS effects	1	2	3	4	5	6	7	8	9	10	Mean	SEM
no PS												
20 min	95.6	99.4	96.8	115.3	115.7	97.8	111.2	96.0	95.5	95.2	**101.85**	**2.72**
40 min	142.3	101.8	102.6	89.6	102.7	97.5	99.4	102.5	100.3	85.4	**102.41**	**4.81**
60 min	143.7	101.6	97.6	102.2	101.8	94.3	100.7	102.5	103.4	94.2	**104.20**	**4.51**
3 J/cm^2 PS												
20 min	125.9	102.3	99.2	98.5	100.9	103.6	100.4	100.2	99.7	91.3	**102.2**	**2.83**
40 min	126.4	106.4	107.9	103.5	106.6	110.3	103.8	106.5	108.9	70.8	**105.11**	**4.33**
60 min	143.8	106.6	110.3	107.7	111.6	106.6	106.6	107.5	108.4	63.2	**107.23**	**6.06**
6 J/cm^2 PS												
20 min	113.3	114.3	82.1	116.4	89.6	117.6	112.2	89.7	86.8	86.6	**100.86**	**4.70**
40 min	112.8	89.8	107.6	115.5	92.5	104.3	109.4	111.9	91.9	87.9	**102.36**	**3.38**
60 min	117.3	73.1	125.5	120.6	89.2	113.1	121.4	122.2	89.5	80.4	**105.23**	**6.29**
9 J/cm^2 PS												
20 min	124.3	125.3	95.1	127.4	100.6	126.6	123.2	100.7	97.8	97.6	**111.86**	**4.54**
40 min	140.6	96.4	148.8	143.9	103.5	142.4	147.7	148.5	105.8	98.7	**127.63**	**7.31**
60 min	157.6	115.4	159.8	160.9	122.5	160.4	161.7	165.5	122.8	115.7	**144.23**	**6.91**
18 J/cm^2 PS												
20 min	132.5	148.1	109.6	95.8	113.8	87.0	116.2	122.5	114.3	122.2	**116.20**	**5.46**
40 min	152.8	96.3	102.6	118.3	114.2	125.6	185.2	133.6	137.6	148.8	**131.50**	**8.34**
60 min	130.9	167.4	125.6	129.6	153.2	220.1	127.1	138.5	157.3	152.3	**150.20**	**9.02**
27 J/cm^2 PS												
20 min	136.5	121.9	114.9	115.9	118.6	49.5	125.6	119.8	100.6	78.8	**108.21**	**8.17**
40 min	143.7	46.1	132.4	121.2	129.9	135.9	139.6	145.2	140.6	120.3	**125.49**	**9.23**
60 min	142.2	193.1	85.2	148.2	127.1	125.9	147.6	164.5	201.9	155.9	**149.16**	**10.63**

	Wilcoxon signed rank test									
	no PS & 3 PS		no PS & 6 PS		no PS & 9 PS		no PS & 18 PS		no PS & 27 PS	
Time	p-value	V	p-value	V	p-value	V	p-value	V	p-value	V
20 min	**0.9593**	27	**0.9594**	28	**0.04685**	8	**0.07446**	10	**0.2026**	15
40 min	**0.3863**	19	**0.8785**	29	**0.02182**	5	**0.01086**	1	**0.07446**	10
60 min	**0.0737**	10	**0.7213**	24	**0.005062**	0	**0.00691**	1	**0.01252**	3

The statistical significance in Supplementary Table 2 was assessed by Wilcoxon signed rank tests (n=10 mice in each group). The statistical tests involved two-sided analyses. Source data are provided as a Source Data file.

Supplementary Table 3 –GNRs level (μg/g tissue) in dcLN in healthy adult mice before and after PS

Number of mouse/ Time of observation of PS effects	1	2	3	4	5	6	7	8	9	10	Mean	SEM
no PS												
20 min	5.7	1.5	1.2	1.3	0.9	0.8	0.7	1.6	1.4	0.1	**1.52**	**0.49**
40 min	8.5	2.6	2.8	3.1	2.2	2.5	2.7	2.6	1.9	0.1	**2.90**	**0.68**
60 min	8.5	3.8	3.4	3.9	3.6	3.1	4.8	5.3	3.5	0.1	**4**	**0.66**
3 J/cm^2 PS												
20 min	4.8	2.5	3.1	2.2	1.4	3.6	1.5	2.7	1.1	0.1	**2.30**	**0.43**
40 min	7.6	3.3	3.1	2.5	3.1	3.2	2.8	2.7	3.6	0.1	**3.20**	**0.58**
60 min	6.6	4.3	3.5	4.5	4.9	4.7	5.2	4.4	4.8	0.1	**4.30**	**0.53**
6 J/cm^2 PS												
20 min	1.3	2.2	2.8	1.4	1.2	2.2	0.8	1.1	2.3	2.1	**1.74**	**0.21**
40 min	0.1	4.5	3.1	2.2	3.9	2.6	0.2	0.4	2.6	3.5	**2.31**	**0.50**
60 min	7.1	0.7	6.8	5.7	0.9	5.9	0.1	0.4	6.9	2.9	**3.74**	**0.95**
9 J/cm^2 PS												
20 min	3.6	4.8	2.4	5.2	4.2	2.7	4.6	14.1	4.3	0.1	**4.60**	**1.16**
40 min	9.6	9.8	9.5	9.3	10.4	9.4	9.7	10.1	10.3	9.9	**9.80**	**0.12**
60 min	12.5	9.6	12.4	11.8	12.4	12.1	10.8	24.1	13.1	8.2	**12.70**	**1.35**
18 J/cm^2 PS												
20 min	5.1	2.8	1.2	2.4	4.9	3.2	4.1	3.9	14.1	6.3	**4.80**	**1.13**
40 min	2.7	18.8	9.7	5.4	3.2	2.1	4.1	5.1	7.4	3.5	**6.20**	**1.58**
60 min	7.3	5.1	4.2	6.2	15.3	18.2	19.5	16.7	19.9	5.6	**11.80**	**2.09**
27 J/cm^2 PS												
20 min	3.5	5.9	2.9	0.8	3.9	2.1	26.7	3.8	1.2	7.2	**5.80**	**2.40**
40 min	5.4	13.4	10.2	5.2	6.8	7.8	8.9	3.8	2.3	8.2	**7.20**	**1.03**
60 min	13.1	8.1	15.7	19.9	16.2	11.8	2.3	17.1	10.2	16.6	**13.10**	**1.64**

	Wilcoxon signed rank test									
	no PS & 3 PS		no PS & 6 PS		no PS & 9 PS		no PS & 18 PS		no PS & 27 PS	
Time	p-value	V	p-value	V	p-value	V	p-value	V	p-value	V
20 min	**0.05061**	6	**0.1688**	14	**0.02088**	3	**0.01086**	1	**0.05934**	9
40 min	**0.2135**	12	**0.8785**	29	**0.005062**	0	**0.05934**	9	**0.0166**	4
60 min	**0.3139**	14	**0.8785**	29	**0.005034**	0	**0.009344**	2	**0.00691**	1

The statistical significance in Supplementary Table 3 was assessed by Wilcoxon signed rank tests (n=10 mice in each group). The statistical tests involved two-sided analyses. Source data are provided as a Source Data file.

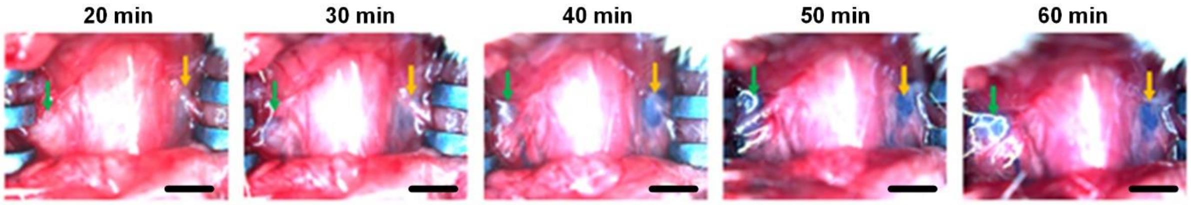

Supplementary Figure 3 – Detection of effective time for fluorescent imaging of EBD (5%, 5µL) accumulation in dcLNs after dye injection into the right lateral ventricle. The green arrow represents the right dcLN, and the yellow arrow represents the left dcLN of the mouse. Scale bar: 2 mm, 3 replicates.

Supplementary Table 4 –EBD fluorescent intensity (arb. units) in dcLN in healthy adult mice before and after PS

Number of mouse/ Time of observation of PS effects	1	2	3	4	5	6	7	8	9	10	Mean	SEM
no PS												
20 min	8.5	1.1	0.3	0.6	0.9	0.8	0.2	0.5	0.4	0.1	**1.34**	**0.80**
40 min	10.5	4.2	5.1	4.8	4.6	2.3	3.2	3.8	4.3	0.2	**4.30**	**0.83**
60 min	16.2	7.5	7.4	5.2	7.3	6.6	6.2	6.1	6.4	3.1	**7.200**	**1.08**
3 J/cm² PS												
20 min	3.1	1.4	1.8	1.2	1.7	2.2	2.4	2.1	1.9	1.2	**1.90**	**0.19**
40 min	11.7	2.4	2.1	2.5	2.4	3.4	2.3	3.5	3.2	0.5	**3.40**	**0.96**
60 min	20.8	4.6	6.2	6.6	4.9	6.4	5.5	6.7	5.9	0.4	**6.80**	**1.66**
6 J/cm² PS												
20 min	1.8	1.9	2.2	2.1	0.7	2.3	2.5	1.7	2.4	1.5	**1.91**	**0.17**
40 min	4.1	8.8	1.3	4.6	4.4	5.1	5.3	4.2	4.5	4.7	**4.70**	**0.57**
60 min	6.9	19.1	6.7	5.5	7.3	0.5	6.5	5.2	7.4	4.9	**7**	**1.49**
9 J/cm² PS												
20 min	7.8	6.9	8.8	8.5	8.3	7.3	8.5	19.3	8.2	4.4	**8.80**	**1.23**
40 min	12.8	12.7	13.4	14.2	13.2	12.3	13.8	24.5	14.4	8.7	**14**	**1.27**
60 min	18.1	21.4	17.4	15.2	17.5	24.2	14.1	23.5	14.3	17.7	**18.34**	**1.14**
18 J/cm² PS												
20 min	7.1	10.1	4.8	3.2	19.7	3.7	11.2	5.4	21.7	6.1	**9.30**	**2.07**
40 min	17.5	24.3	8.2	8.7	6.3	7.1	15.9	16.3	5.8	18.9	**12.90**	**2.04**
60 min	27.5	18.1	6.5	17.6	34.1	9.8	18.3	5.4	29.5	25.2	**19.20**	**3.12**
27 J/cm² PS												
20 min	10.1	17.2	3.4	6.8	7.5	4.1	6.2	2.4	5.7	15.6	**7.90**	**1.58**
40 min	14.2	26.5	8.1	9.6	7.4	12.3	10.6	6.1	12.4	9.8	**11.70**	**1.82**
60 min	12.1	16.2	7.4	28.4	58.2	5.7	13.4	8.2	32.1	7.3	**18.90**	**5.22**

	Wilcoxon signed rank test									
	no PS & 3 PS		no PS & 6 PS		no PS & 9 PS		no PS & 18 PS		no PS & 27 PS	
Time	p-value	V	p-value	V	p-value	V	p-value	V	p-value	V
20 min	**0.07427**	10	**0.0926**	11	**0.00691**	1	**0.00691**	1	**0.005062**	0
40 min	**0.1394**	42	**0.4752**	20.5	**0.005034**	0	**0.005062**	0	**0.005062**	0
60 min	**0.3863**	36	**0.9527**	22	**0.005062**	0	**0.01252**	3	**0.0284**	4

The statistical significance in Supplementary Table 4 was assessed by Wilcoxon signed rank tests (n=10 mice in each group). The statistical tests involved two-sided analyses. Source data are provided as a Source Data file.

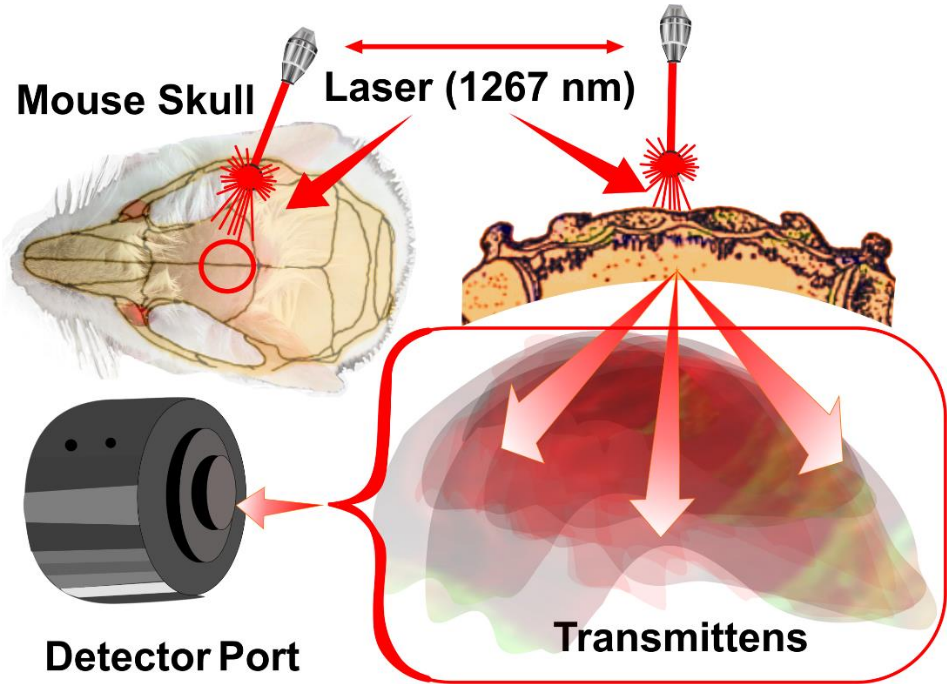

Supplementary Figure 4 - Scheme of the optical setup for measuring the mouse scalp and skull transmittance level for 1267 nm QD semiconductor laser. The optical setup was developed with the 1267 nm laser (Innolume Gmbh, Germany) and collimator (Thorlabs Ltd., USA) given 5 mm diameter beam on the illuminated surface of the mouse skull. The scattering coefficient and diffuse transmittance were measured on the fresh mouse scalp and the skull of 10 mice.

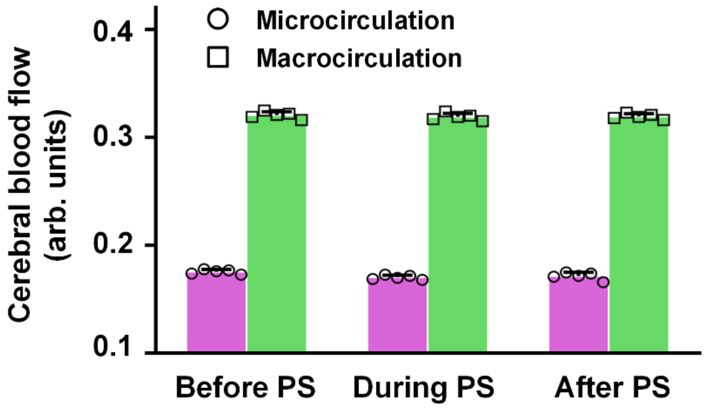

Supplementary Figure 5 - The changes in the cerebral blood flow (CBF) detected by laser speckle contrast imaging before and after PS in single dose 9 J/cm^2 in adult healthy mice. All values are presented as Mean ± SEM. Wilcoxon tests were used (n=5 mice in each group). The statistical tests involved two-sided analyses. Source data are provided as a Source Data file.

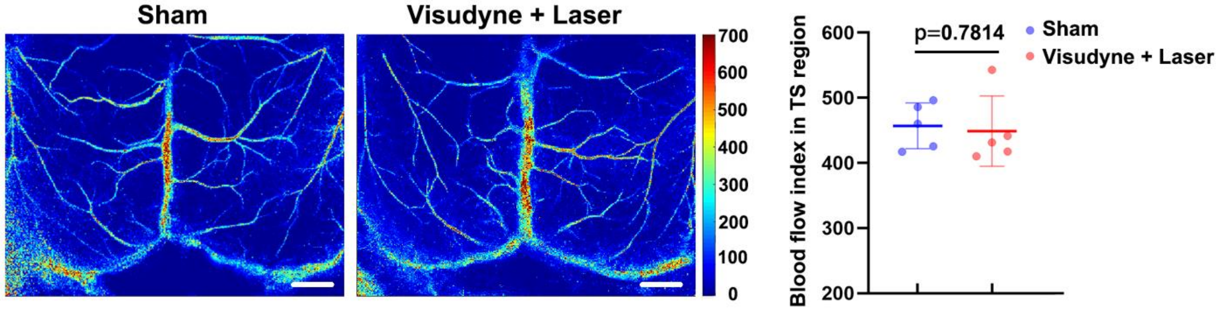

Supplementary Figure 6 – The changes in the CBF detected by laser speckle imaging in the sham group and after photodynamic ablation of MLVs in the Visudyne+Laser group. Scale bar: 1 mm. There were no changes in CBF after photodynamic effects (P=0.7814, n=5 mice in each group). All values are presented as Mean ± SEM. The independent-samples T tests were used. The statistical tests involved two-sided analyses. Source data are provided as a Source Data file.

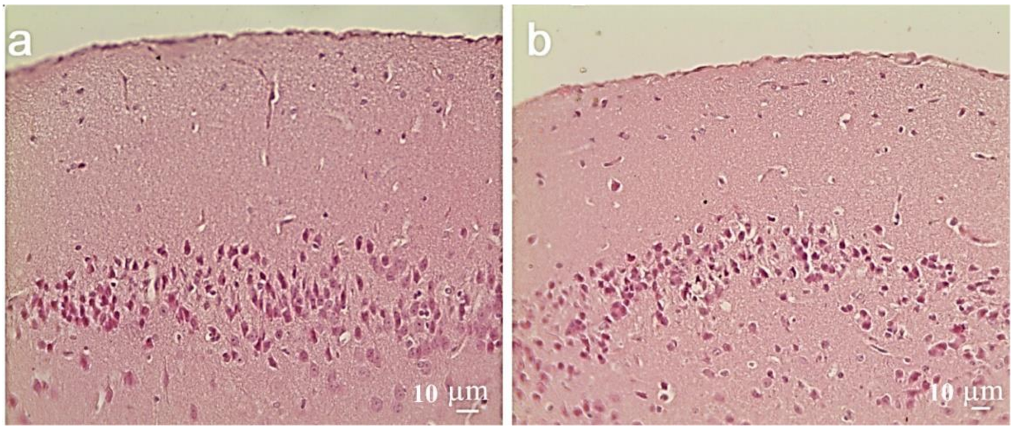

Supplementary Figure 7 – The histological analysis (H&E) of the cortex; (a) before and **(b)** after the PS course 4 J/cm^2 in newborn rats. Scale bar: 10 µm. 3 replicates.

Supplementary Table 5 - Temperature (°C) at the external surface of skull and the brain cortex before and after PS in newborn rats

Number of mouse/ Thermocouple positioning	1	2	3	4	5	6	7	8	9	10	Mean	SEM
no PS												
The skull external surface	35.82	36.00	36.04	36.12	36.08	35.71	36.03	36.01	35.73	36.19	**35.973**	**0.052**
Under the skull on the cortex surface	37.18	37.15	37.12	37.21	37.12	37.17	37.10	37.16	37.19	37.14	**37.154**	**0.011**
4 J/cm^2 PS												
The skull external surface	36.06	36.11	36.16	36.00	36.17	36.21	36.10	36.18	36.14	36.11	**36.124**	**0.020**
Under the skull on the cortex surface	37.07	37.10	37.12	37.18	37.06	37.20	37.16	37.15	37.08	37.07	**37.119**	**0.016**

Wilcoxon signed rank test (**no PS** & **4 J/cm^2 PS**)	
**Thermocouple positioning**	**p-value**
The skull external surface	**0.0414**
Under the skull on the cortex surface	**0.0850**

The statistical significance in Supplementary Table 5 was assessed by Wilcoxon signed rank tests (n=10 mice in each group). The statistical tests involved two-sided analyses. Source data are provided as a Source Data file.

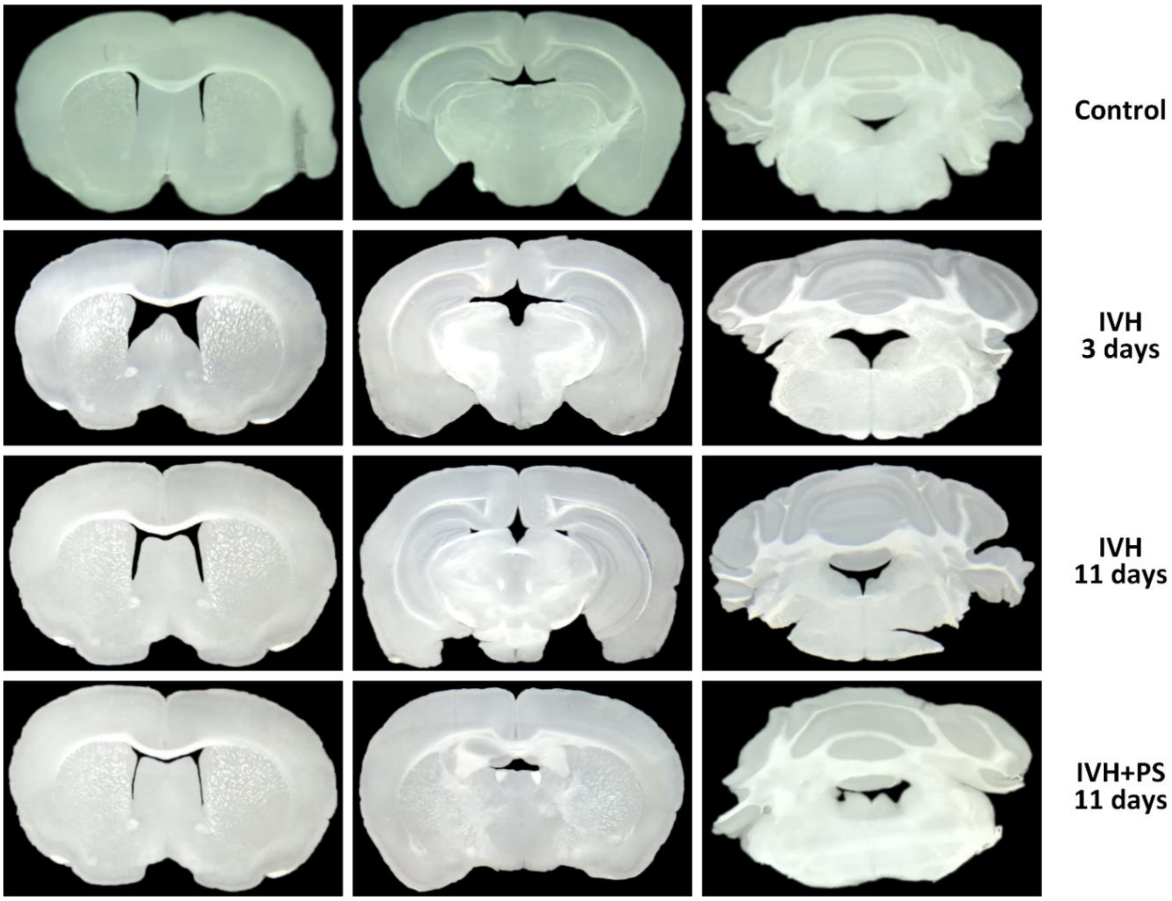

Supplementary Figure 8 – 2D confocal images of fresh brains illustrating size of the right lateral ventricle; (three projections) in 4-day old newborn rats in the control (sham) group, 3 days and 11 days after IVH without and after PS-course 4 J/cm^2. 3 replicates.

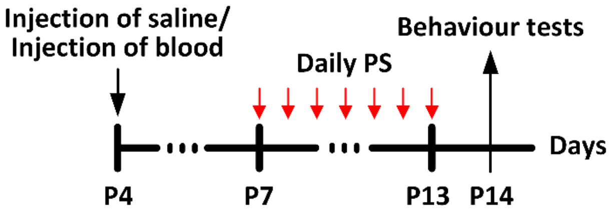

Supplementary Figure 9 – Schematic illustration of time of performance of behavior tests in pups.

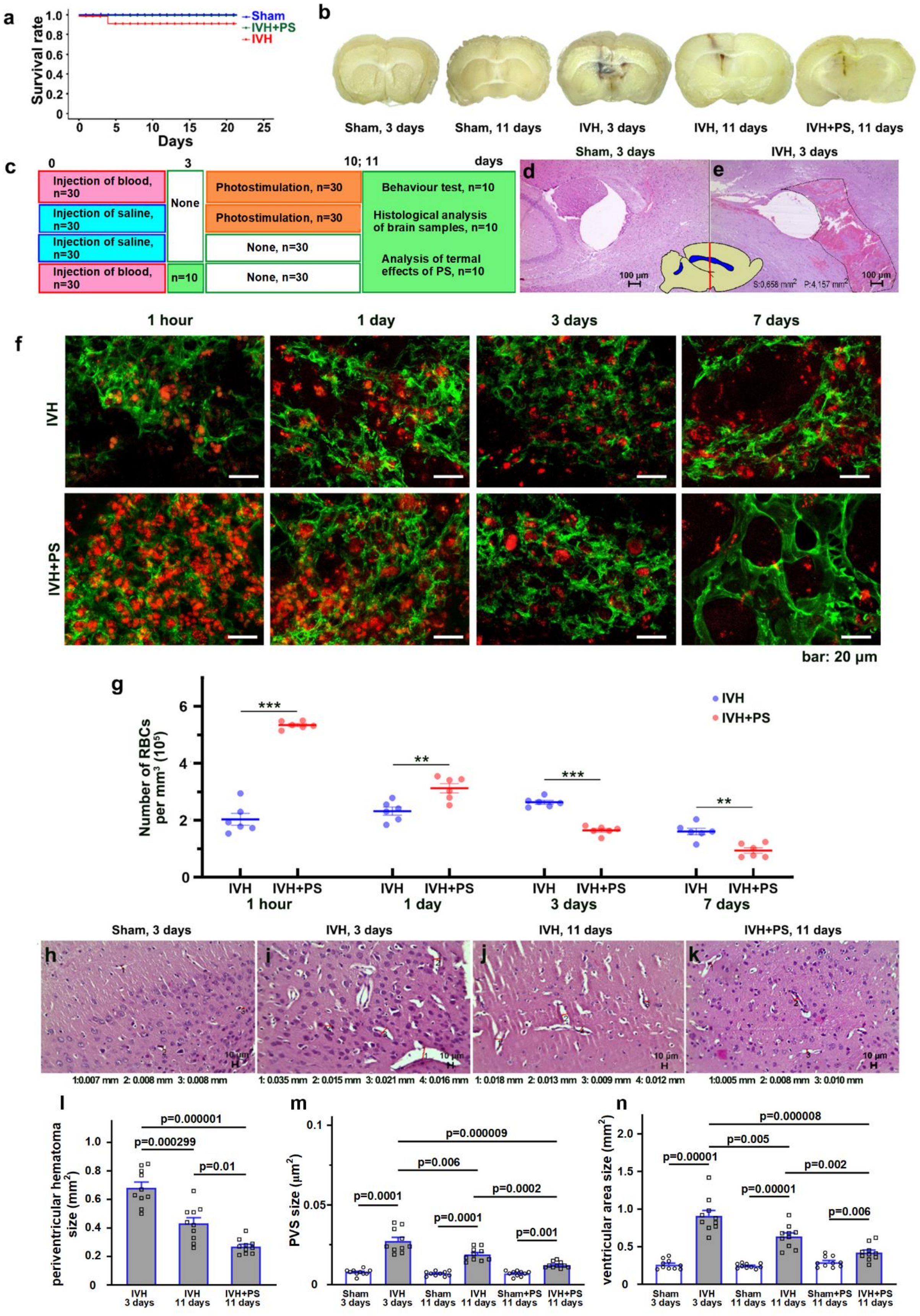

Supplementary Figure 10 – Therapeutic effects of the PS-9 J/cm^2 course in adult mice with IVH. (**a**) Kaplan-Meier overall survival plots in the tested groups with and without PS-course. The number of surviving mice by day 21 of observation was not significantly changed in the IVH+PS

group than in the IVH group (n=20 mice in each group. P=0.161, X2 test Log Rank (Mantel-Cox)=1.961, Kaplan-Meier method). (**b**) Representative 2D images (from 3 replicates) of the normal brain. (**c**) The schematic diagram of time points of experiments and the number of animals in the experimental groups. (**d** and **e**) Representative histological imaging (from 3 replicates) of the normal brain tissues around the right later ventricle and the periventricular hematoma formed 3 days after IVH (Scale bar: 100 μm). (**f**) Representative confocal images (from 2 replicates) of presence of RBC in dcLNs of mice without and after the PS-course (Scale bar: 20 μm). (**g**) The number of RBCs in dcLN 1 hour, 1 day, 3 days and 7 day after IVH with/without PS (n=6 mice in each group). (**h-k**) Histological images illustrating the vasogenic edema in brain tissue of normal (h); 3 days after IVH (i); 11 days after IVH (j) and 11 days IVH+PS (k) (Scale bar: 10 μm, n=3 independent experiments). (**l-n**) The quantitative analysis of periventricular hematoma (l), PVS size (m), and ventricular area size (n) on 3 days and 11 days after IVH with and without the PS-course (n=10 mice in each group). All values are presented as Mean ± SEM. Independent-samples T tests were used for **g**; and Welch's tests were used for **l-n**. The statistical tests involved two-sided analyses. **P < 0.01 and ***P < 0.001. Source data are provided as a Source Data file.

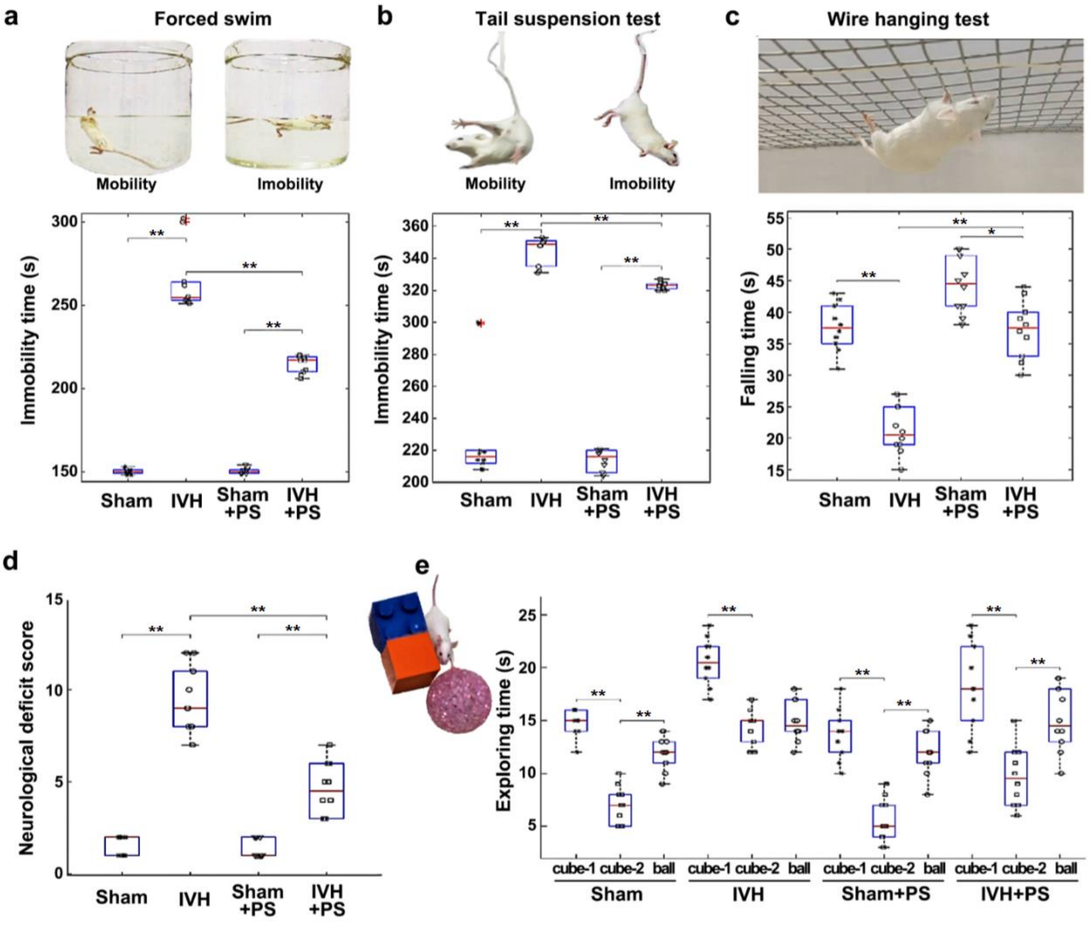

Supplementary Figure 11 – The PS effects on the locomotor and memory functions. (**a** and **b**) The immobility time (sec) in the forced (**a**) and tail (**b**) tests in sham mice and in mice with IVH without and after the PS-course (n=10 mice in each group). (**c**) The falling latency time (sec) in the wire hanging test in sham mice and in mice with IVH without and after the PS-course (n=10 mice in each group). (**d**) The neurologic deficit score measured by a 24-point scoring system in sham mice and in mice with IVH without and after the PS-course (n=10 mice in each group). (**e**) The time of exploring of the new and old objects in the novel object recognition test in sham mice and in mice with IVH without and after the PS-course (n=10 mice in each group). The box indicates the upper and lower quantiles, the thick line in the box indicates the median and whiskers indicate 2.5th and 97.5th percentiles. All values are presented as Mean ± SEM. The Wilcoxon tests were used for **a-e**. The statistical tests involved two-sided analyses. *P < 0.05 and **P < 0.01. Source data are provided as a Source Data file.

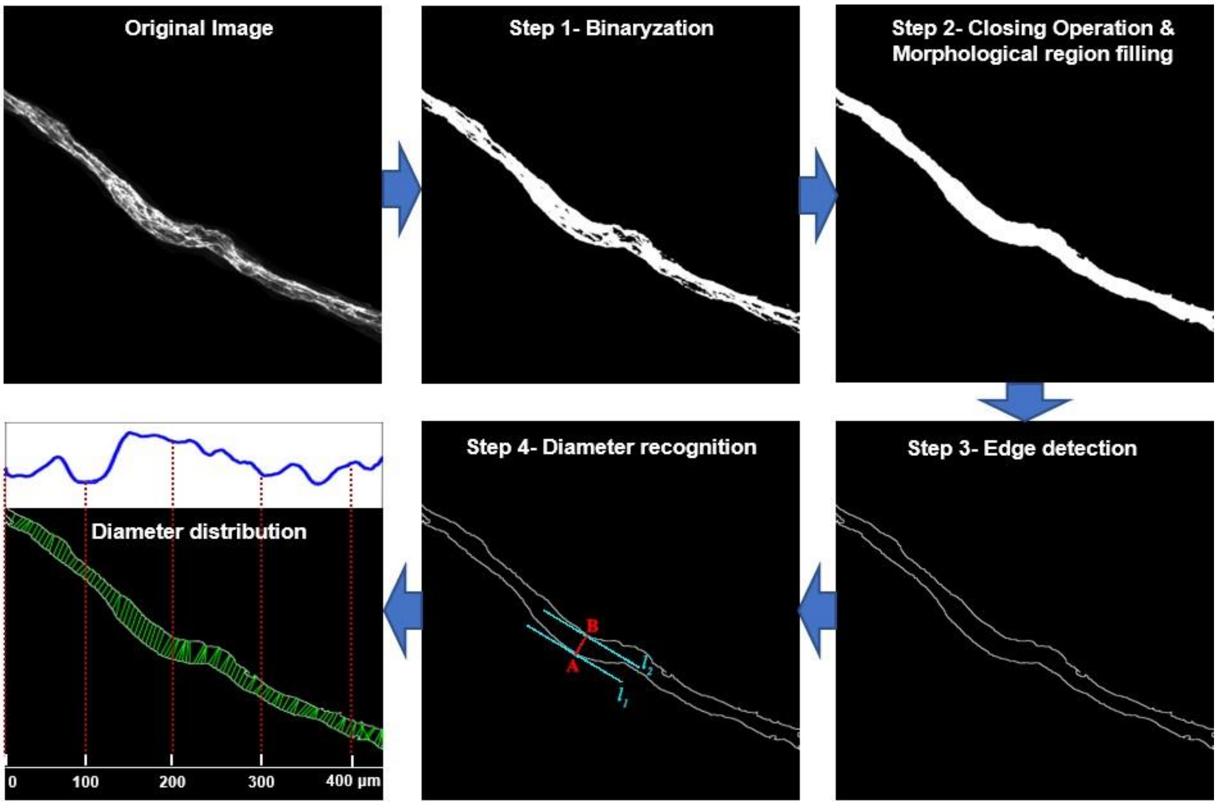

Supplementary Figure 12 – Schematic illustration of MLV diameter distribution calculation.
