# Supplementary material for: Photostimulation of brain lymphatics in male newborn and adult rodents for therapy of intraventricular hemorrhage
Source: Nat Commun. 2023 Sep 29;14:6104. doi: 10.1038/s41467-023-41710-y (PMC10541888; doi:10.1038/s41467-023-41710-y)
Supplement: Supplementary file 3 — Description of additional supplementary files [file 41467_2023_41710_MOESM3_ESM.pdf]

Description of Additional Supplementary Files Document

File name: Supplementary Movie 1
Description: The lymphatic contraction before photostimulation.

File name: Supplementary Movie 2
Description: The lymphatic contraction after photostimulation.
