# Supplementary material for: Photostimulation of brain lymphatics in male newborn and adult rodents for therapy of intraventricular hemorrhage
Source: Nat Commun. 2023 Sep 29;14:6104. doi: 10.1038/s41467-023-41710-y (PMC10541888; doi:10.1038/s41467-023-41710-y)
Supplement: Supplementary file 6 — Reporting Summary [file 41467_2023_41710_MOESM6_ESM.pdf]

nature portfolio

Corresponding author(s): Dan Zhu, Oxana Semyachkina-Glushkovskaya, Tingting Yu

Last updated by author(s): Sep 14, 2023

Reporting Summary

Nature Portfolio wishes to improve the reproducibility of the work that we publish. This form provides structure for consistency and transparency in reporting. For further information on Nature Portfolio policies, see our Editorial Policies and the Editorial Policy Checklist.

Statistics

For all statistical analyses, confirm that the following items are present in the figure legend, table legend, main text, or Methods section.

n/a	Confirmed	
☐	☒	The exact sample size (*n*) for each experimental group/condition, given as a discrete number and unit of measurement
☐	☒	A statement on whether measurements were taken from distinct samples or whether the same sample was measured repeatedly
☐	☒	The statistical test(s) used AND whether they are one- or two-sided<br>*Only common tests should be described solely by name; describe more complex techniques in the Methods section.*
☒	☐	A description of all covariates tested
☐	☒	A description of any assumptions or corrections, such as tests of normality and adjustment for multiple comparisons
☐	☒	A full description of the statistical parameters including central tendency (e.g. means) or other basic estimates (e.g. regression coefficient) AND variation (e.g. standard deviation) or associated estimates of uncertainty (e.g. confidence intervals)
☐	☒	For null hypothesis testing, the test statistic (e.g. *F*, *t*, *r*) with confidence intervals, effect sizes, degrees of freedom and *P* value noted<br>*Give P values as exact values whenever suitable.*
☒	☐	For Bayesian analysis, information on the choice of priors and Markov chain Monte Carlo settings
☒	☐	For hierarchical and complex designs, identification of the appropriate level for tests and full reporting of outcomes
☐	☒	Estimates of effect sizes (e.g. Cohen's *d*, Pearson's *r*), indicating how they were calculated

Our web collection on statistics for biologists contains articles on many of the points above.

Software and code

Policy information about availability of computer code

Data collection	The commercial softwares used in this study for data collection are: Zen 2011 SP2 (Version 8.0.0.273, Carl Zeiss GmbH), NIS-Elements AR (Version 4.20, Nikon).
Data analysis	The commercial softwares used in this study for data analysis are: Imaris (Version 7.6, Bitplane AG), SPSS (Version 26, IBM), Matlab (Version 2014a, Mathworks).<br>The open source software used in this study to analyze the data is: ImageJ (Version 1.51j8, NIH).<br>The custom-made code: The code used to analyze these data is freely available on Github [https://github.com/HUST-LAB/MLV-diameter].

For manuscripts utilizing custom algorithms or software that are central to the research but not yet described in published literature, software must be made available to editors and reviewers. We strongly encourage code deposition in a community repository (e.g. GitHub). See the Nature Portfolio guidelines for submitting code & software for further information.

Data

Policy information about availability of data

All manuscripts must include a data availability statement. This statement should provide the following information, where applicable:

- Accession codes, unique identifiers, or web links for publicly available datasets
- A description of any restrictions on data availability
- For clinical datasets or third party data, please ensure that the statement adheres to our policy

The data supporting the findings of this study are available within the article, the Supplementary Information files and the Source Data files that accompany this article. Source Data are provided with this paper.

Research involving human participants, their data, or biological material

Policy information about studies with human participants or human data. See also policy information about sex, gender (identity/presentation), and sexual orientation and race, ethnicity and racism.

Reporting on sex and gender	The human studies were performed on 3 male patients.
Reporting on race, ethnicity, or other socially relevant groupings	*Please specify the socially constructed or socially relevant categorization variable(s) used in your manuscript and explain why they were used. Please note that such variables should not be used as proxies for other socially constructed/relevant variables (for example, race or ethnicity should not be used as a proxy for socioeconomic status).*<br>*Provide clear definitions of the relevant terms used, how they were provided (by the participants/respondents, the researchers, or third parties), and the method(s) used to classify people into the different categories (e.g. self-report, census or administrative data, social media data, etc.)*<br>*Please provide details about how you controlled for confounding variables in your analyses.*
Population characteristics	The human studies were performed on 3 male patients (average age 42, n=3: 51, 37, 40) died from IVH (parenchymatous-ventricular hemorrhage in the right cerebral hemisphere with formation of subdural and intracerebral hematomas with blood rupture into ventricles and subarachnoid space).
Recruitment	The species of human meninges at autopsy were obtained from the Department of Pathological Anatomy at the Saratov Medical State University. The present study was performed according to a protocol approved by the Committee of Science and Research Ethics, the Saratov Medical State University. All personal data are stored in strict ethical control, and samples were coded before the analyses of tissue. In accordance with GOST R ISO 14155-2014 (Russian National Standard) of the Ministry of Health of the Russian Federation (Article 38 of the Federal Law dated November 21, 2011) and the Order of the Ministry of Health of the Russian Federation No. 354 dated June 6, 2013 “On the procedure for conducting pathological and anatomical studies” is not required the informed consent from died patients or their relatives for the analysis of the brains and organs to establish the cause of death. The study was carried out on material in the amount necessary to make a post-mortem diagnosis and nothing additional was taken from the corpse.
Ethics oversight	The present study was performed according to a protocol approved by the Committee of Science and Research Ethics, the Saratov Medical State University.

Note that full information on the approval of the study protocol must also be provided in the manuscript.

Field-specific reporting

Please select the one below that is the best fit for your research. If you are not sure, read the appropriate sections before making your selection.

☒ Life sciences ☐ Behavioural & social sciences ☐ Ecological, evolutionary & environmental sciences

For a reference copy of the document with all sections, see nature.com/documents/nr-reporting-summary-flat.pdf

Life sciences study design

All studies must disclose on these points even when the disclosure is negative.

Sample size	Sample size was determined according to previously publications and the number of biological replicates necessary for ensuring statistical significance. Please see the figure legends for details.
Data exclusions	No data were excluded from the data analysis.
Replication	Experiments were repeated in at least 2 independent experiments with similar results. All experiments were reproduced to reliably support the conclusions stated in the manuscript. The replication details are in the corresponding figure legends.
Randomization	All samples were randomly allocated into experimental groups.
Blinding	The investigators were blinded to group allocation during data collection and analysis.

Reporting for specific materials, systems and methods

We require information from authors about some types of materials, experimental systems and methods used in many studies. Here, indicate whether each material, system or method listed is relevant to your study. If you are not sure if a list item applies to your research, read the appropriate section before selecting a response.

Materials & experimental systems

n/a	Involved in the study
☐	☒ Antibodies
☒	☐ Eukaryotic cell lines
☒	☐ Palaeontology and archaeology
☐	☒ Animals and other organisms
☒	☐ Clinical data
☒	☐ Dual use research of concern
☒	☐ Plants

Methods

n/a	Involved in the study
☒	☐ ChIP-seq
☒	☐ Flow cytometry
☒	☐ MRI-based neuroimaging

Antibodies

Antibodies used	Primary antibodies: rat Alexa Fluor 488-conjugated anti-LYVE-1 (Cat. No. FAB2125G, R&D Systems, dilution 1:500); rabbit anti-PROX-1(Cat. No. ab 101851, Abcam, dilution 1:500); rat Alexa Fluor 647-conjugated anti-CD-31(Cat. No. 102416, BioLegend, dilution 1:500); rabbit anti- LYVE-1(Cat. No. ab 218535, Abcam, ilution 1:500); rabbit anti-LYVE-1 (Cat. No. ab219556, Abcam, dilution 1:500); mouse anti-CD-31 (Cat. No. ab187377, Abcam, dilution 1:500); mouse anti-glycophorin A (GPA) (Cat. No. ab7503, Abcam, dilution 1:500); rabbit anti-CLDN 5 (Cat. No. ab217316, Abcam, dilution 1:500); mouse anti-GFAP (Cat. No. ab279290, Abcam, dilution 1:500).<br>Secondary antibody: goat anti-rabbit IgG (H+L) Alexa Fluor 555 (Cat. No. A21429, Invitrogen, dilution 1:500); anti-rabbit IgG (H+L) Alexa Fluor 488 goat (Cat. No. A11008, Invitrogen, dilution 1:500); goat anti-mouse IgG (H+L) Alexa Four 555 (Cat. No. A21422, Invitrogen, dilution 1:500,); goat anti-mouse IgG (H+L) Alexa Fluor 647 (Cat. No. A21235, Invitrogen, dilution 1:500).
Validation	The rat Alexa Fluor 488-conjugated anti-LYVE-1 (FAB2125G, R&D Systems) has been validated by the manufacturer: R&D Systems, https://www.rndsystems.com/cn/search?keywords=FAB2125G%2C%20.<br>The rabbit anti-Prox-1 (ab101851, Abcam) has been validated by the manufacturer: Abcam, https://www.abcam.cn/products/primary-antibodies/prox1-antibody-bsa-and-azide-free-ab101851.html.<br>The rat Alexa Fluor 647-conjugated anti-CD-31(102416, BioLegend) has been validated by the manufacturer: BioLegend, https://www.biolegend.com/en-us/products/alexa-fluor-647-anti-mouse-cd31-antibody-3092.<br>The rabbit anti- LYVE-1(ab 218535, Abcam) has been validated by the manufacturer: Abcam, https://www.abcam.cn/products/primary-antibodies/lyve1-antibody-epr21771-ab218535.html.<br>The rabbit anti-LYVE-1 (ab219556, Abcam) has been validated by the manufacturer: Abcam, https://www.abcam.cn/products/primary-antibodies/lyve1-antibody-epr21857-ab219556.html.<br>The mouse anti-CD-31 (ab187377, Abcam) has been validated by the manufacturer: Abcam, https://www.abcam.cn/products/primary-antibodies/cd31-antibody-c313-ab187377.html.<br>The mouse anti-glycophorin A (GPA) (ab7503, Abcam) has been validated by the manufacturer: Abcam, https://www.abcam.cn/products/primary-antibodies/glycophorin-a-antibody-jc159-prediluted-ab7503.html.<br>The rabbit anti-CLDN 5 (ab217316, Abcam) has been validated by the manufacturer: Abcam, https://www.abcam.cn/products/primary-antibodies/claudin-5-antibody-ab217316.html.<br>The mouse anti-GFAP (ab279290, Abcam) has been validated by the manufacturer: Abcam, https://www.abcam.cn/products/primary-antibodies/gfap-antibody-epr1034y--mouse-igg2a-chimeric-ab279290.html.

Animals and other research organisms

Policy information about studies involving animals; ARRIVE guidelines recommended for reporting animal research, and Sex and Gender in Research

Laboratory animals	Male BALB/c mice (25-28 g, 2-3 months) and male postnatal days 4 newborn Wistar rats (7-8 g) were used in this study. All animals were maintained under specific pathogen-free conditions under controlled temperature (18-22 °C) and humidity (50–60%) and a 12-h dark/12-h light cycle (7a.m. to 7p.m.), with access to regular rodent's chow and sterilized tap water ad libitum.
Wild animals	The study did not involve wild animals.
Reporting on sex	Male Balb/c and male newborn Wistar rats were used in this study.
Field-collected samples	The study did not involve samples collected from the field.
Ethics oversight	The experimental protocols were approved by the Local Bioethics Commission of the Saratov State University (Protocol No. 7); Experimental Animal Management Ordinance of Hubei Province, P. R. China (No. 1000639903375); the Institutional Animal Care and Use Committee of the University of New Mexico, USA (#200247).

Note that full information on the approval of the study protocol must also be provided in the manuscript.
